# The effect of adolescent social isolation on vulnerability for methamphetamine addiction behaviours in female rats

**DOI:** 10.1007/s00213-022-06103-x

**Published:** 2022-03-26

**Authors:** Paige I. Webb, Timothy J. Hill, Nicholas A. Everett, Jade L. Thornton, Jennifer L. Cornish, Sarah J. Baracz

**Affiliations:** 1 Department of Psychology, C3A, Macquarie University, North Ryde, NSW 2109 Australia; 2 Centre for Emotional Health, Macquarie University, North Ryde, NSW 2109 Australia

**Keywords:** Stress, Adolescence, Methamphetamine, Addiction, Females, Social isolation

## Abstract

**Rationale:**

Stress exposure during adolescence contributes to developing a methamphetamine (METH) use disorder. However, most of the studies investigating addiction-related behaviours include only male rodents, despite METH addiction rates being higher in females. Furthermore, animal studies investigating the effects of stress on methamphetamine addiction have used only basic self-administration models which may not be sensitive to the effects of stress.

**Objectives:**

This project explored whether adolescent isolation stress exposure increases the incidence of four key addiction-related behaviours in female rats.

**Methods:**

Thirty-two female rat pups were caged in groups of four or individually during adolescence from postnatal (PND) day 22, with the latter being re-socialised in groups of four on PND 43. In adulthood, rats were tested for addiction-like behaviours in a METH self-administration paradigm modelling motivation to take METH, persistence in drug-seeking behaviour when METH was not available, resistance to extinction, and propensity to reinstate after a period of withdrawal.

**Results:**

Adolescent social isolation resulted in lower METH intake during acquisition; however, the paradigm modelling drug-seeking when the drug was unavailable engendered intermittent METH bingeing in all rats, abolishing the group differences in intake during this phase. Adolescent social isolation also accelerated extinction of non-reinforced lever pressing, and increased stress-primed reinstatement, compared to the group-housed rats.

**Conclusions:**

Adolescent social isolation stress alters various methamphetamine addiction-like behaviours in female rats.

## Introduction

The psychostimulant methamphetamine (METH) is a highly addictive drug that is frequently abused on a global scale. In Australia, METH abuse has increased threefold since 2010, whereby per capita METH use is now higher than almost any other country (National Ice Taskforce 2015). Methamphetamine use rates have also steadily increased in the USA, whereby in 2017, an estimated 964,000 people aged 12 years or older were suffering from a METH use disorder, up from 684,000 people in 2016 (National Institute on Drug Abuse 2021). Prolonged or repeated METH use can lead to a range of severe mental and physical health issues, ranging from anxiety, depression, aggression and cognitive dysfunction, to cardiac and respiratory failure (Topp et al. 2002; Darke et al. 2008; Cruickshank and Dyer 2009; McKetin et al. 2016). The issues surrounding METH use disorder are worsened by the lack of effective pharmacological or psychological interventions (Lee and Rawson 2008).

Substance use disorder, including METH use, is characterised by maladaptive and compulsive drug use patterns that occur in a small proportion of drug users (Wagner and Anthony 2002). The drug use patterns characterising stimulant use disorder as defined by the Diagnostic and Statistical Manual-5 (DSM-5) include a high motivation to take the drug, persistence in drug-seeking behaviour or not being able to stop using the drug, and craving the drug, triggering relapse to drug-taking after a period of abstinence (American Psychiatric Association 2013). These three addiction behaviours can be tested in rodent models of voluntary drug intake, the most common being the self-administration procedure (Brown et al. 2011). The inclusion of these behaviours in a single paradigm allows for greater translatability from rodent models to human behaviour, which is advantageous over the more commonly used self-administration paradigms, which may only measure one or two behaviours characterising substance use disorder (Deroche-Gamonet et al. 2004). The use of self-administration paradigms that measure numerous behaviours relevant to substance use disorder allows for a more comprehensive investigation into the genetic factors and environmental influences that contribute to the development of substance use disorder, including experiences with, and responses to, chronic stress (Hynes et al. 2018).

The likelihood of developing METH use disorder is strongly influenced by exposure to stressful experiences. This is particularly pronounced when stress exposure occurs during critical developmental periods (Lupien et al. 2009). Adolescence is one such sensitive period of development, during which social interaction, social acceptance and social support greatly influence cognitive schemas and behavioural patterns, which can contribute to the emergence of psychopathology. For example, social isolation during adolescence can result in the development of maladaptive coping strategies and is associated with higher rates of anxiety (Walker et al. 2019) and depression (Jahng et al. 2012), as well as increased rates of drug-taking behaviour (Mumtaz et al. 2018). A review of the long-term effects of social isolation during adolescence in rodents similarly indicates an increase in anxiety-like behaviours, as well as an increase in drug reward sensitivity, even after subsequent resocialisation in adulthood (Walker et al. 2019). In studies where social isolation increases drug-taking behaviour in rodents, the isolation period typically extends from weaning through to adulthood (Ding et al. 2005; Fosnocht et al. 2019; Hofford et al. 2015), preventing an examination of the impact of social isolation stress when specifically suffered during adolescence.

Negative consequences from either adolescent stress exposure or METH use occur in both sexes, yet are heightened in females. After adolescent social isolation, females display increased depression-like behaviours relative to their group-housed counterparts (Jahng et al. 2012) and increased anhedonia-like responses compared to males (Hong et al. 2012). Females without a history of adolescent stress exposure who are exposed to METH experience a faster transition to dependence on METH (Cox et al. 2013; Riley et al. 2018; Shen et al. 2014) and a higher incidence of addiction-related behaviours compared to their male counterparts (Cox et al. 2013). In addition, female, but not male, rats display higher behavioural sensitisation to amphetamine, a behavioural model of the neurobiological changes induced by repeated drug exposure, after adolescent chronic stress exposure (Hynes et al. 2018). Despite the recognition that females are impacted by adolescent stress more so than males, a recent review of behavioural development following adolescent social isolation reported that the majority of research in this area has only been conducted with male subjects, with only 25% of the included studies measuring behaviour in a female sample (Walker et al. 2019). As such, greater investigation of the impact of adolescent stress exposure on vulnerability to develop a METH use disorder in females is essential.

In order to reduce the vulnerability to develop METH use disorder in females, a better understanding of the negative environmental factors affecting susceptibility for METH use disorder is needed. The current study implemented a model of social isolation that is specific to the key developmental period of adolescence. An intravenous self-administration (IVSA) paradigm was then used in adulthood, with schedules modelling the key characteristics of stimulant use disorder as defined by the DSM-5: (1) persistence in drug-seeking, measured as either drug seeking during periods of signalled drug unavailability or as resistance to behavioural extinction; (2) motivation to take METH, measured by progressive ratio reinforcement; and (3) propensity to reinstate to drug-seeking behaviour when exposed to stimuli that trigger relapse in humans (Brown et al. 2011). Additionally, this study focused on the impact of adolescent social isolation on vulnerability for METH use disorder in female rats, the understudied sex. We predicted that female rats exposed to a social isolation stress during adolescence would demonstrate stronger engagement in addiction-relevant behaviours, which would be indicative of increased vulnerability for METH use disorder, compared to a control group of socially housed females.

## Materials and methods

### Animals

Eight, time-mated Long Evans pregnant rats were sourced from the Animal Resource Centre (Perth, WA, Australia) and arrived at our facility on gestation day 16. Dams were housed individually in large cages (63 × 40 × 31 cm) with chow and water available ad libitum. Dams birthed 32 female and 30 male pups. Dams and their respective litters were left undisturbed until weaning on postnatal day (PND) 22.

For the duration of the experiment, animals were housed in a humidity- and temperature-controlled (21 + / − 1 °C) holding room on a 12-h light–dark cycle (lights on at 06:00). The study was approved by the Macquarie University Animal Ethics Committee and was conducted in accordance with the Australian Code of Practice for the Care and Use of Animals for Scientific Purposes (8th Edition, 2013).

### Adolescent social isolation

**Fig. 1:**
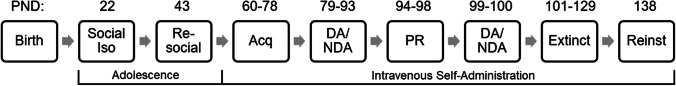
Experimental timeline of social isolation and METH intravenous self-administration. *PND*, postnatal day; *Social Iso*, social isolation; *Re-social*, resocialisation; *Acq*, acquisition; *DA/NDA*, drug available/non-drug available; *PR*, progressive ratio; *Extinct*, extinction; *Reinst*, reinstatement

On PND22, considered early adolescence (Lupien et al. 2009), pups were weaned from dams and 2 to 4 female offspring per litter were randomly allocated to either the isolation-housed (adolescent stress) or group-housed (control) condition and were matched for weight, see Fig. 1. Isolation-housed females were individually housed in small cages (46 × 32 × 27 cm), whilst group-housed female rats were housed with three littermates (Jahng et al. 2012) in large cages (63 × 40 × 31 cm) (Yorgason et al. 2013; Hofford et al. 2015). Environmental enrichment (e.g. Perspex domes, straws) was consistently provided across conditions. On PND43, considered mid-adolescence (Lupien et al. 2009), isolated pups were re-socialised and caged in groups of four (Whitaker et al. 2012; Lukkes et al. 2009) with rats from unique litters. Rats were then left undisturbed until early adulthood. Male pups were not included in this experiment.

### Drugs

Methamphetamine hydrochloride (purity 99%; METH, ‘ice’) was sourced from the Australian Government Analytical Laboratories (Pymble, NSW). For IVSA, METH was dissolved in saline (0.9%) at a dose of 0.1 mg/kg per 0.05 mL infusion and filtered through a Millipore syringe filter (0.45 µm). For intraperitoneal (IP) injection, METH was administered at a dose of 1 mg/kg and at a volume of 1 mL/kg. Yohimbine hydrochloride was sourced from Tocris Bioscience (Bristol, UK), was dissolved in distilled water at a dose of 0.625 mg/kg or 1.25 mg/kg and was administered at a volume of 2 mL/kg.

### Catheter implantation surgery

In early adulthood (PND55-59), rats underwent surgery for permanent catheter implantation into the right jugular vein. Catheter construction, implantation and post-operative care were conducted as previously described (Baracz et al. 2016). Rats were given 5 to 7 days to recover before experimental procedures began.

### Intravenous METH self-administration

#### Apparatus

Rats learnt to self-administer METH in standard sound-attenuated operant chambers (Med Associates, VT, USA). Each chamber had two retractable levers, a cue light above each lever, a house light and a tone generator. Each lever was programmed as either active or inactive and their position was counterbalanced across chambers. Four infrared photobeam detectors were also positioned on the sidewall of each operant chamber to measure locomotor activity. Active and inactive lever presses, number of infusions and locomotor activity were recorded using MED-PC-V software (Med Associates). Locomotor activity was recorded during all addiction-relevant stages, except for persistence in drug-seeking when the drug was signalled not available.

#### Procedure

Prior to each session, catheters were flushed with 0.1 mL heparin solution (60 IU) and at the end of each session were flushed with 0.2 mL heparinised (60 IU) cephazolin solution. Self-administration sessions lasted 3 h, unless otherwise stated. Sessions were held 7 days a week, with the exception of acquisition when sessions were run 5 days a week. To prevent overdosing, a 20-s time out was enforced after each infusion, during which no further infusions could be received, and the maximum number of infusions per session was limited to 120. The session ended either when 3 h elapsed, or 120 infusions of METH had been delivered.

#### Acquisition of intravenous self-administration

Starting in early adulthood (PND60; Lupien et al. 2009), rats were trained to self-administer intravenous METH on a fixed-ratio one (FR-1) schedule of reinforcement. On this schedule, one active lever press resulted in a METH infusion and a compound cue comprising of illumination of the cue light and emission of a distinct 78 dB 5 kHz tone for 5 s. Depression of the inactive lever was recorded but had no programmed consequences. Rats were considered to have acquired METH IVSA when the ratio of active to inactive lever presses reached at least 2:1 per session and a minimum of 10 infusions were earned each session for the last three sessions (Hicks et al. 2016).

#### Persistence in drug-seeking during signalled drug unavailability

Following 14 days on the FR-1 schedule, the IVSA sessions were divided into alternating 40-min drug-available (DA) and 20-min non-drug available (NDA) periods, in line with the procedure used by Brown et al. (2011). During DA periods, a constant 78-dB white noise was emitted, except during the 20-s timeout after an infusion. Similar to the FR-1 scheduled sessions, receiving an infusion resulted in illumination of the cue light and generation of the 78 dB 5 kHz tone for 5 s. The NDA periods were differentiated from the DA periods by the illumination of the house light and termination of the white noise. During the NDA periods, depression of either lever had no programmed consequence. After stable responding on an FR-1 schedule, rats progressed to the fixed-ratio three (FR-3) schedule, followed by the fixed-ratio five (FR-5) schedule. Once stable, responding on the FR-5 schedule was achieved; rats completed five consecutive test DA/NDA sessions, followed by a sixth test DA/NDA session after progressive ratio (PR) testing (Brown et al. 2011). Stable responding was considered active lever pressing within 15% across three consecutive sessions. The sixth DA/NDA test session was included in the paradigm to return rats to a fixed-ratio schedule before extinction training commenced.

#### Motivation to take METH

Following the fifth DA/NDA test session, rats were trained on a PR schedule, which required an increasing number of responses on the active lever to receive an infusion of METH following the function: response ratio (nearest integer) = 5e ^(injection number × 0.2) − 5^ (Richardson and Roberts 1996). This required a response on the active lever of 1, 2, 4, 6, 9, 12, 15, 20, 25 and 32 depressions and so on and provides an estimate of how motivated rats were for an infusion of METH. The break point was recorded for each rat in each of these sessions. The break point was the response requirement at which rats were no longer willing to work for the drug during the 3-h IVSA session. During PR–scheduled sessions, cue exposure was identical to acquisition sessions and the DA period in the DA/NDA sessions. After stable responding was achieved, rats completed three PR test sessions.

#### Extinction

Rats were subsequently exposed to protracted behavioural extinction for 29 days. Depression of either lever was recorded but did not result in drug infusion or cue exposure. Extinction sessions were initially 3 h in length (extinction day 1) and then were progressively reduced to 2 h (extinction day 2) and then 1-h sessions (extinction day 3 onwards). Rats received a saline IP injection on the final extinction day to habituate rats to the injection procedure and minimise any effects of injection stress on behaviour during testing sessions.

#### Reinstatement

Following behavioural extinction, rats underwent three types of reinstatement tests: cue-induced, METH-primed and yohimbine (stress)-primed reinstatement. Yohimbine is a pharmacological stressor which increases noradrenaline release and elicits reinstatement to drug-seeking behaviour in rats and craving in humans (Shepard et al. 2004; Moran-Santa Maria et al. 2014). All reinstatement test sessions lasted 1 h. Each reinstatement test was separated by 2 or 3 extinction days. Rats did not proceed to the next test day unless the extinction criterion of < 10 active presses in the previous extinction session was achieved.

For cue-induced reinstatement, rats were re-exposed to the compound cue (cue-light and tone) associated with METH. At the beginning of the session, one non-contingent compound cue was presented for 30 s. Thereafter, each subsequent active lever press resulted in exposure to the compound cue. Rats then underwent two METH-primed reinstatement sessions, whereby rats received an injection of either saline (0.9%) or METH (1 mg/kg, IP) immediately prior to being placed in the operant chamber. Next, rats were exposed to three yohimbine-primed reinstatement sessions. Rats received an injection of either distilled water or 1 of 2 doses of yohimbine hydrochloride (0.625 mg/kg or 1.25 mg/kg) 30 min prior to being placed in the operant chamber. The conditions of the METH- and yohimbine-primed reinstatement sessions were identical to that of extinction sessions.

### Statistical analysis

Data are displayed as the mean ± SEM and were analysed using IBM SPSS (version 27) with significance set at *p* < 0.05. Data pertaining to acquisition, persistence in drug-seeking and motivation to take METH were analysed using a mixed model analysis of variance (ANOVA) with drug-taking day as the within-subject factor and adolescent stress condition as the between-subject factor. For both extinction and reinstatement, mixed model ANOVAs were used in addition to one-way ANOVAs to compare adolescent stress conditions on the rate of extinction and propensity to relapse, respectively. To determine whether rats reinstated, paired samples *t*-tests were undertaken for each condition comparing the extinction day prior or vehicle test session to the reinstatement test session. Additionally, for the first FR1 DA/NDA session and the last FR5 DA/NDA session, infusion data was analysed for binge-like intake. A ‘binge’ was operationalised as at least 5 infusions in 5 min as seen previously (Belin et al. 2009). The number of binges, average number of infusions per binge and proportion of infusions taken in a binge were compared between conditions and across the period of DA/NDA training using mixed model and one-way ANOVAs. Lastly, simple linear regressions were used to determine whether locomotor activity on day 1 of METH self-administration predicted lifetime METH intake; active lever pressing on PR; NDA and cue-, drug- and stress-induced reinstatement or binge-like intake.

In cases where the assumption of equal variance was not met, Greenhouse Geisser corrections are reported. For all analyses, litter was included as a covariate.

## Results

Of the original 32 rats, 31 rats completed the study. One rat, from the group-housed condition, was excluded due to not acquiring METH IVSA. Additionally, locomotor data is missing for 2 rats due to technical difficulties and from 1 rat during acquisition due to being an outlier. Lastly, data for 3 rats is missing from the binge analysis for the last FR-5 session due to a technical problem with the Med-Associates file.

### Acquisition of METH self-administration

**Fig. 2:**
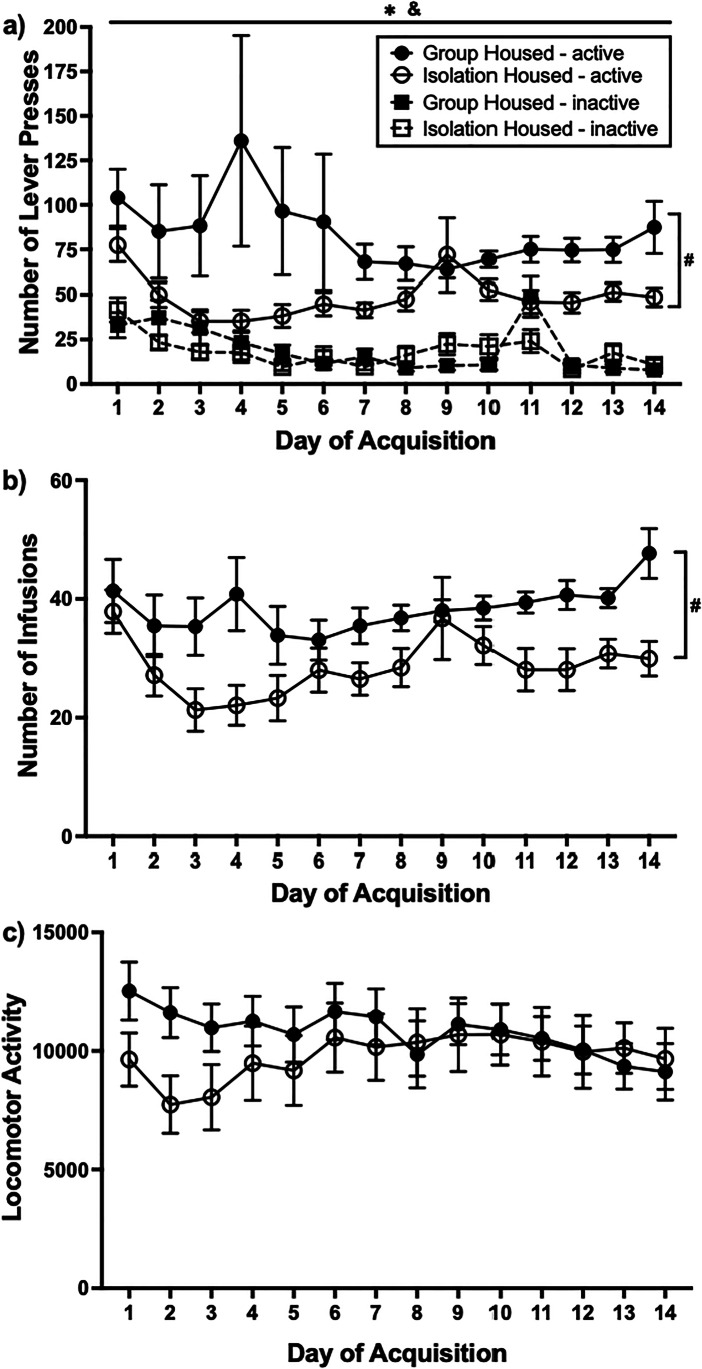
The effect of adolescent housing condition on acquisition of METH IVSA. Group-housed rats took more METH during the 14-day acquisition period. Mean (± SEM) active and inactive lever presses (**a**), infusions (**b**) and locomotor activity (**c**) during acquisition. *p < 0.05 significant main effect of day, #*p* < 0.05 significant main effect of adolescent housing condition, &*p* < 0.05 significant main effect of lever

Rats acquired METH IVSA, as indicated by a significant increase in METH-paired active lever pressing (*F*(13,364) = 2.853, *p* < 0.001; Fig. 2a). A significant difference between active and inactive lever responses was also apparent across the 14-day period, indicating rats differentiated between the active and inactive lever (*F*(1364) = 6.914, *p* = 0.014). Whilst there was no significant difference in infusions across the acquisition period, the number of infusions was high from day 1 (mean 38.77, SEM 3.231) to day 14 (mean 39.06, SEM 2.858; F(13,364) = 1.728, *p* = 0.054; Fig. 2b). As the number of infusions remained high across acquisition, locomotor activity did not differ across the 14-day period (*F*(5.627, 140.678) = 1.570, *p* = 0.165; Fig. 2c).

When comparing acquisition of METH-taking behaviour across adolescent housing conditions, rats in the group-housed condition demonstrated significantly greater active lever responding than rats in the isolation-housed condition (*F*(1.28) = 5.202, *p* = 0.030). Group-housed rats also took a significantly higher number of METH infusions compared to isolation-housed rats across the acquisition period (*F*(1.28) = 9.384, *p* = 0.005), indicating that METH intake during acquisition was greater in group-housed rats relative to isolation-housed rats. Lastly, there was no difference between conditions on inactive lever pressing (*F*(1.28) = 0.645, *p* = 0.429) or on locomotor activity (*F*(1.25) = 0.648, *p* = 0.428), indicating no differences between groups on overall activity during these sessions. In addition, locomotor activity on day 1 of METH self-administration did not significantly predict lifetime METH intake; active lever pressing on PR; NDA and cue-, drug- and stress-induced reinstatement or binge-like intake as assessed with a linear regression (all *p* > 0.05).

### Persistence in drug-seeking during the NDA period

**Fig. 3:**
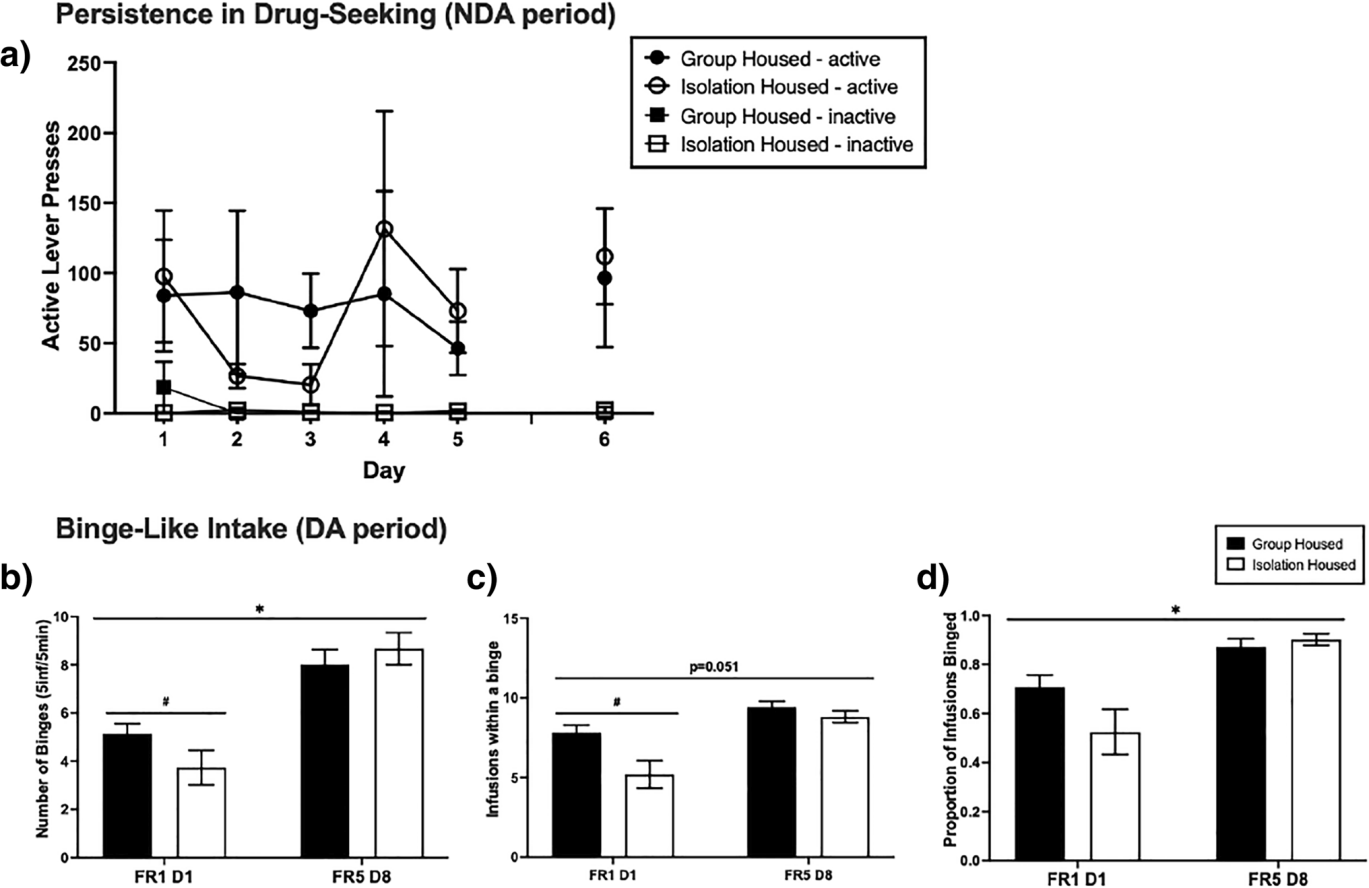
The effect of adolescent housing condition on persistence in drug seeking during non-drug available (NDA) periods and binge-like METH intake during drug available (DA) periods. Lever pressing on the NDA period did not differ by condition; however, patterns of METH bingeing increased in isolation-housed rats over time to be no different to the group-housed rats. Mean (± SEM) active lever presses during the NDA periods (**a**), and number of binges (**b**), number of infusions within a binge (**c**) and proportion of infusions binged (**d**) during the DA periods. *p < 0.05 significant main effect of day, #*p* < 0.05 significant main effect of adolescent housing condition. On the sixth DA/NDA session conducted after PR, there was no significant difference between group-housed and isolation-housed rats on active lever pressing during the NDA periods (*F*(1.28) = 0.059, *p* = 0.810)

Across the five consecutive DA/NDA FR-5 test sessions, active lever pressing did not fluctuate (*F*(4112) = 0.871, *p* = 0.484; Fig. 3a). Active lever pressing across rats was however highly variable, due to a subset of rats continuing to press during the NDA period whilst the majority did not. Importantly, the proportion of rats that continued to press during the five DA/NDA sessions did not differ by adolescent housing condition (*F*(1.28) = 0.010, *p* = 0.920). Due to this variability, active lever pressing did not significantly differ to inactive lever pressing (*F*(1.28) = 1.504, *p* = 0.230).

### Binge-like intake during the DA period

On the first FR-1 DA/NDA session after acquisition, the pattern of lever pressing for METH differed by adolescent housing condition, whereby the group-housed rats had a higher number of binges than isolation-housed rats (*F*(1.28) = 4.540, *p* = 0.042; Fig. 3b). Additionally, the average number of infusions within a binge was higher in the group-housed rats relative to the isolation-housed rats (*F*(1.28) = 7.138, *p* = 0.012; Fig. 3c). The proportion of infusions taken in a binge did not differ by condition (*F*(1.28) = 3.354, *p* = 0.078; Fig. 3d). From the first DA/NDA FR-1 session to the last FR-5 session prior to PR, the number of binges significantly increased (*F*(1.25) = 7.226, *p* = 0.013), as did the proportion of infusions taken in a binge (*F*(1.25) = 4.362, *p* = 0.047). The average binge size across the two timepoints was close to reaching significance (*F*(1.25) = 4.213, *p* = 0.051). Surprisingly, on the last FR-5 DA/NDA session, the number of binges did not differ by adolescent housing condition (*F*(1.25) = 0.007, *p* = 0.935). Additionally, the average binge size (*F*(1.25) = 1.111, *p* = 0.302) and the proportion of infusions taken in a binge (*F*(1.25) = 0.655, *p* = 0.426) did not differ by adolescent housing condition. This demonstrates that across the DA/NDA sessions, the isolation-housed rats increased their drug-taking to a level equivalent to that of the group-housed rats.

### Motivation to take METH

**Fig. 4:**
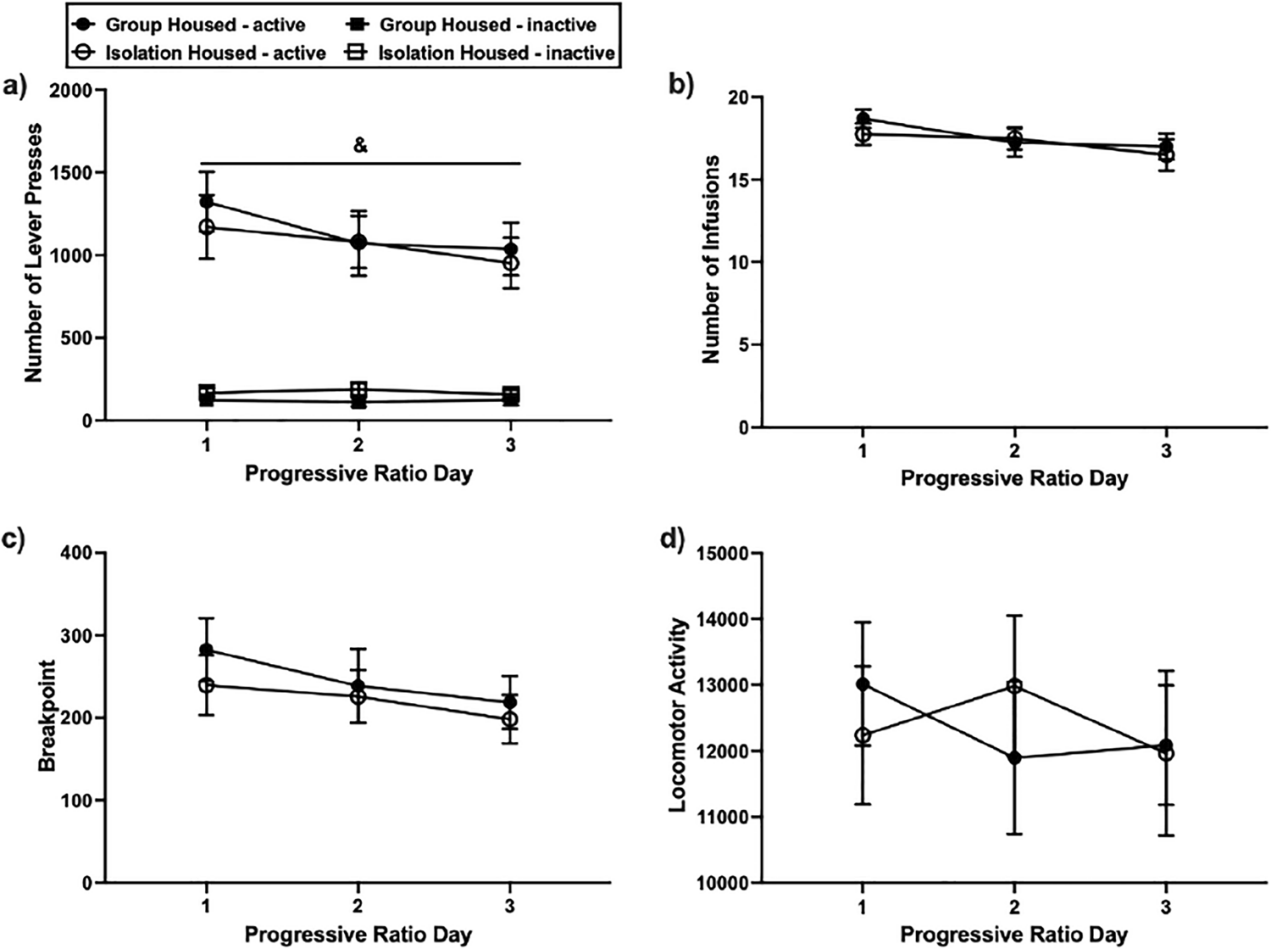
The effect of adolescent housing condition on motivation to take METH. Drug-taking behaviour did not differ by condition. Mean (± SEM) active and inactive lever presses (**a**), infusions (**b**), breakpoint (**c**) and locomotor activity (**d**) on progressive ratio. &*p* < 0.05 significant main effect of lever

Across the three PR test sessions, active lever pressing was significantly higher than inactive lever pressing (*F*(1.28) = 9.619, *p* = 0.004; Fig. 4a). Across these sessions, group- and isolation-housed rats performed equivalent active lever presses (*F*(1.28) = 0.200, *p* = 0.658), received an equivalent number of infusions (*F*(1.28) = 0.245, *p* = 0.624; Fig. 4b), reached a similar break point (*F*(1.28) = 0.299, *p* = 0.589; Fig. 4c) and demonstrated similar levels of locomotor activity (*F*(1.26) = 0.014, *p* = 0.907; Fig. 4d).

### Lifetime METH intake

**Fig. 5:**
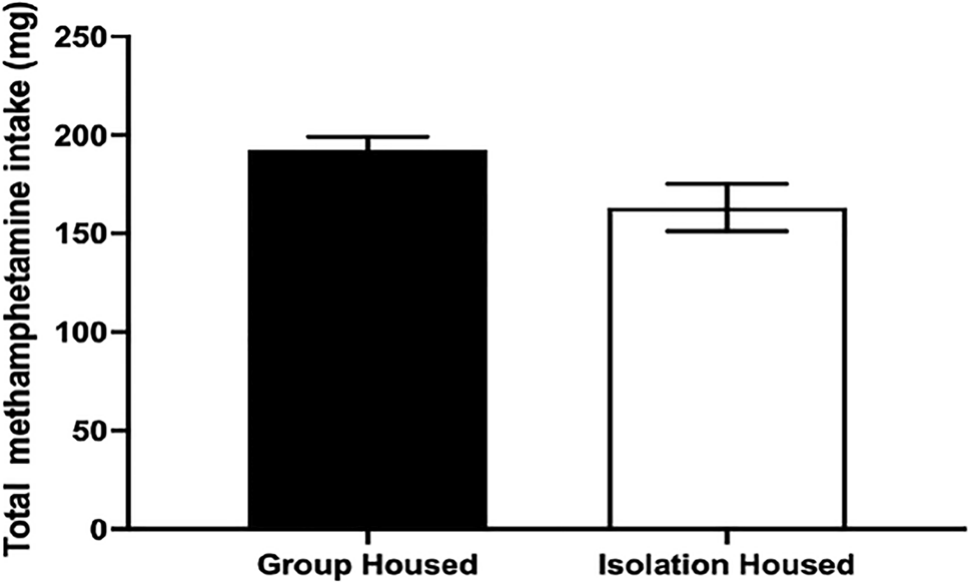
The effect of adolescent housing condition on lifetime METH intake. Group-housed and isolation-housed rats took a similar amount of METH across the duration of the experiment. Mean (± SEM) METH intake

Over the course of the experiment, rats in the group-housed and isolation-housed conditions took an equivalent amount of METH (*F*(1.28) = 4.027, *p* = 0.055; Fig. 5).

### Extinction

**Fig. 6:**
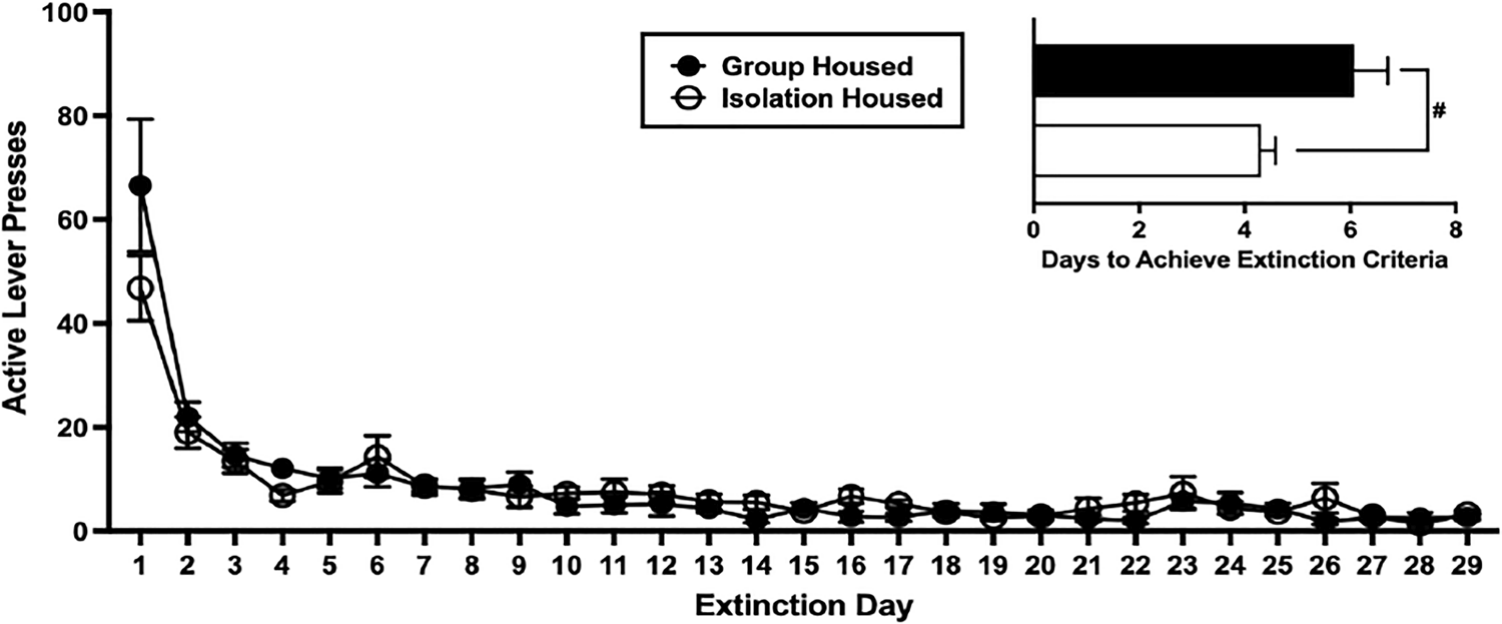
The effect of adolescent housing condition on extinction from METH-taking. Group-housed rats took longer to extinguish METH-taking behaviour than isolation-housed rats. Inset graph depicts the average number of days to extinguish active lever pressing. Mean (± SEM) active lever presses during extinction. #*p* < 0.05 significant main effect of adolescent housing condition

Active lever responding significantly decreased from the first (day 1 mean 57, SEM 7) to the last day of extinction (day 29 mean 3, SEM 0.5; *F*(2.718, 76.104) = 8.834, *p* < 0.001; Fig. 6). Rats in the isolation-housed condition extinguished active lever pressing (< 20 active lever presses during three consecutive extinction sessions) on average by day 4, which was significantly quicker than rats in the group-housed condition, who on average reached this criteria by day 6 (*F*(1.28) = 6.357, *p* = 0.018).

### Reinstatement

#### Cue-induced reinstatement

**Fig. 7:**
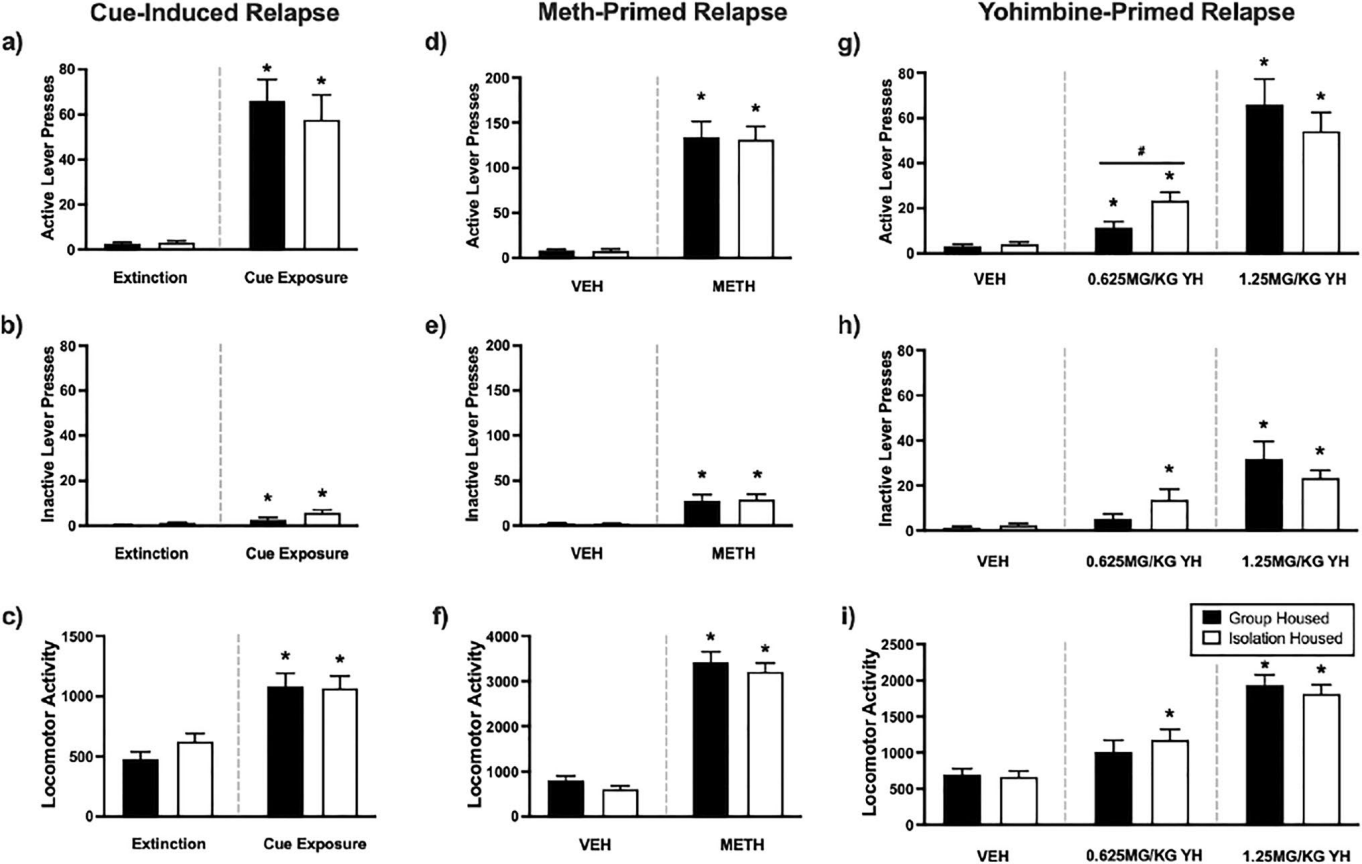
The effect of adolescent housing condition on cue-, METH- and yohimbine-primed reinstatement. Isolation-housed rats showed a stronger propensity to relapse after a 0.625-mg/kg yohimbine prime relative to group housed rats. Mean (± SEM) active lever presses (**a**, **d**, **g**), inactive lever presses (**b**, **e**, **h**) and locomotor activity (**c**, **f**, **i**) on reinstatement tests. *p < 0.05 vs. respective extinction or vehicle session, #*p* < 0.05 significant main effect of adolescent housing condition. On the cue test session, rats differentiated between the active and inactive lever *(F*(1.28) = 5.995, *p* = 0.021). However, when comparing conditions, no significant difference on active lever responding (*F*(1.28) = 0.308, *p* = 0.584), inactive lever presses (*F*(1.28) = 3.577, *p* = 0.069) or locomotor activity (*F*(1,28) = 0.188, *p* = 0.668) was evident

Rats from both conditions reinstated to drug-seeking behaviour following presentation of a METH-compound cue, as indicated by a significant increase in active lever responding on the cue-induced relapse session compared to the extinction day prior (group-housed: *t*(14) =  − 6.681, *p* < 0.001; isolation-housed: *t*(15) =  − 4.855, *p* < 0.001; Fig. 7a). Additionally, inactive lever presses (group-housed: *t*(14) =  − 2.555, *p* = 0.023; isolation-housed: *t*(15) =  − 3.674, *p* = 0.002; Fig. 7b) and locomotor activity (group-housed: *t*(14) =  − 4.248, *p* = 0.001; isolation-housed: *t*(15) =  − 2.719, *p* = 0.016; Fig. 7c) were significantly higher on the relapse session relative to the extinction day prior.

#### METH-primed reinstatement

Active lever responding was significantly higher during the METH-primed test session compared to the vehicle test session for both conditions (group-housed: *t*(14) =  − 7.450, *p* < 0.001; isolation-housed: *t*(15) =  − 8.946, *p* < 0.001; Fig. 7d). Similarly, inactive lever presses (group-housed: *t*(14) =  − 3.184, *p* = 0.007; isolation-housed: *t*(15) =  − 4.502, *p* < 0.001; Fig. 7e) and locomotor activity (group-housed: *t*(14) =  − 11.097, *p* < 0.001; isolation-housed: *t*(15) =  − 8.861, *p* < 0.001; Fig. 7f) were significantly higher on the relapse session relative to the extinction day prior.

On the METH-primed test session, rats pressed the active lever more than the inactive lever (*F*(1.28) = 15.436, *p* = 0.001). When comparing conditions, there was no significant difference between group-housed and isolation-housed rats on active lever responding (*F*(1.28) = 0.003, *p* = 0.955), locomotor activity (*F*(1.26) = 0.650, *p* = 0.427) or inactive lever pressing (*F*(1.28) = 0.593, *p* = 0.448).

#### Yohimbine-primed reinstatement

Active lever pressing significantly differed by yohimbine dose (*F*(1.168, 31.534) = 5.502, *p* = 0.021). As such subsequent analyses were conducted for each dose.

For the 0.625mg/kg dose, active lever pressing was significantly higher on the test session relative to vehicle for both conditions (group-housed: *t*(14) =  − 3.321, *p* = 0.005; isolation-housed: *t*(15) =  − 5.274, *p* < 0.001; Fig. 7g). Inactive presses (Fig. 7h) and locomotor activity (Fig. 7i) were significantly higher on the test session relative to vehicle for the isolation-housed condition (inactive presses: *t*(15) =  − 3.315, *p* = 0.005; locomotor activity: *t*(15) =  − 4.446, *p* < 0.001) but not the group-housed condition (inactive presses: *t*(14) =  − 2.116, *p* = 0.053; locomotor activity: *t*(14) =  − 1.999, *p* = 0.069). On the test session, rats pressed the active lever more than the inactive lever (*F*(1.28) = 11.376, *p* = 0.002). When comparing conditions, rats in the isolation-housed condition pressed the active lever significantly more than the group-housed rats, demonstrating a greater propensity to relapse (*F*(1.28) = 6.296, *p* = 0.018). Both locomotor activity (*F*(1.26) = 0.331, *p* = 0.570) and inactive lever pressing (*F*(1.28) = 2.192, *p* = 0.150) were similar across conditions.

For the 1.25mg/kg dose, rats from both conditions pressed the active lever significantly more on the test session relative to vehicle (group-housed: *t*(14) =  − 5.703, *p* < 0.001; isolation-housed: *t*(15) =  − 6.185, *p* < 0.001). Similarly, inactive lever pressing (group-housed: *t*(14) =  − 4.441, *p* < 0.001; isolation-housed: *t*(15) =  − 6.355, *p* < 0.001) and locomotor activity (group-housed: *t*(14) =  − 10.459, *p* < 0.001; isolation-housed: *t*(15) =  − 11.376, *p* < 0.001) increased on the 1.25-mg/kg yohimbine test session for both conditions. On the test session, active lever pressing was significantly higher than inactive pressing (*F*(1.28) = 29.323, *p* < 0.001). When comparing conditions, rats in the group-housed condition pressed the active lever at a similar rate to the isolation-housed condition (*F*(1.28) = 0.679, *p* = 0.417), had similar activity levels (*F*(1.28) = 0.609, *p* = 0.442) and did not differ in their inactive lever pressing (*F*(1.28) = 0.014, *p* = 0.906).

## Discussion

The primary aim of this study was to explore the impact of adolescent social isolation stress on the vulnerability to develop METH addiction behaviours in female rats. We found that rats in the isolation-housed condition self-administered less METH during acquisition than group-housed rats. Upon progression to DA/NDA sessions, isolation-housed rats initially demonstrated reduced bingeing behaviour relative to the group-housed rats, consistent with their METH intake during acquisition. However, by the last DA/NDA session, isolation- and group-housed rats engaged in comparable bingeing behaviour. This similarity in METH intake continued across the PR sessions, such that lifetime METH use across conditions became equivalent. Whilst reinstatement behaviour following cue exposure or a drug prime did not differ by housing condition, isolation-housed rats extinguished drug-seeking behaviour faster, and showed greater stress-induced reinstatement to drug-seeking behaviour than group-housed rats. Overall, these data indicate that adolescent social isolation in females can influence METH-taking, extinction and reinstatement behaviours into adulthood, albeit at times in the opposite direction as was hypothesised.

During the first stage of METH exposure, the acquisition FR1 period, it appears that isolation-housed rats showed greater sensitivity to the psychomotor effects of METH. This was demonstrated through equivalent METH-hyperactivity between isolated- and group-housed rats, despite isolated rats having lower METH intake. This suggests that stress exposure during such a critical period for development could have increased dopamine sensitivity to METH, such that they required a smaller dose of METH to reach similar stimulatory effects. This is in keeping with previous work that has demonstrated heightened dopamine signalling in the nucleus accumbens, a key region in the brain for motivation and reward seeking, after adolescent social isolation (Karkhanis et al. 2016; Yorgason et al. 2013). Additionally, changes to the dopamine system after adolescent social isolation have been associated with locomotor hypersensitivity to psychostimulants. Exposure to adolescent social isolation increased the excitatory presynaptic release probability and intrinsic excitability of medium spiny neurons in the nucleus accumbens, as well as locomotor hypersensitivity to METH in male mice (Zhang et al. 2019). Female rats exposed to adolescent social isolation have also shown an increased locomotor response to an acute injection of diethylpropion, an amphetamine-like drug, relative to group-housed controls, alongside increased dopamine transporter and tyrosine hydroxylase immunocontent and decreased dopamine receptor 2 immunocontent in the dorsal striatum (Lampert et al. 2017). Altogether, these data suggest that rodents socially isolated during adolescence could possibly be more responsive to psychostimulant effects due to changes in the dopamine system.

We found that isolation-housed female rats self-administered less METH on a FR-1 continuous reinforcement schedule and engaged in less binge-like intake when compared to rats group-housed in adolescence, possibly indicating a lower hedonic set point caused by adolescent isolation. Conversely, adolescent housing had no impact on the NDA or PR tasks of compulsive and highly motivated intake, respectively. One explanation is that the hedonic and motivational aspects of METH self-administration are distinct. This is supported by the findings of James et al. (2019) who used behavioural economic procedures to show that the hedonic set-point in rats for cocaine is not necessarily linked to a heightened motivational state, as measured by progressive ratio responding. In agreement with James et al. (2019), our data may therefore support the notion that drug wanting and liking are neither interchangeable nor controlled by the same neural systems (Berridge and Robinson, 2016). It is therefore plausible that adolescent social isolation may disrupt the neural circuits which regulate hedonic set-points, whilst sparing the systems which regulate drug motivation, although this is speculative and requires further research.

Another explanation for the discrepancy between the effects of housing on FR-1 consumption and NDA/PR is that the NDA and PR tests occurred after training on DA/NDA procedures, which closely resemble the intermittent access model developed by Zimmer et al. (2012), which has been shown to exacerbate binge-like intake and various addiction-like behaviours, when compared to continuous drug access. Therefore, instead of uncovering phenotypic differences between housing conditions, the present training on this DA/NDA model may have exacerbated addiction-like behaviour, masking potential phenotypic differences caused by adolescent isolation. In support of this, prior to the DA/NDA phase, adolescent isolated rats had consumed significantly less METH than group-housed rats but after DA/NDA and PR training, this isolation effect on intake was no longer apparent. Furthermore, on the first FR-1 DA/NDA session, isolation-housed rats engaged in less binge-like behaviour than group-housed rats, but after 8 days of FR-5 DA/NDA training, this housing effect was absent, as the number of binges, size of binges and proportion of drug taken within a binge were all increased. In agreement with this explanation, others have found that differences in cocaine self-administration between sign- and goal-tracking rats were subsequently abolished following intermittent access to cocaine (Kawa et al. 2016), further indicating that intermittent access procedures may obscure differences between groups by exacerbating addiction-like behaviours. It is worth considering however, that our DA/NDA model differs somewhat from the well-validated intermittent access model (Zimmer et al. 2012), as our model involved longer DA periods (40 min vs. 5 min) and fewer DA/NDA cycles (3 vs. 12), so a direct comparison cannot be made. Additionally, intravenous METH has a substantially longer half-life than cocaine, approximately 63–75 min in rats, (Rivière et al. 1999; Milesi-Hallé et al. 2005) making it very unlikely that brain concentrations of METH during the 20-min NDA periods approached zero, as is likely the case in Zimmer et al. (2012). Despite this, our intermittent access-like DA/NDA procedure still resulted in an increase in binge-like intake in both housing conditions, and predominantly in isolation-housed rats (Fig. 3b, c, d). This suggests that restricted access, in the absence of plummeting brain drug levels, can engender binge-like intake, although this requires a continuous access group to confirm that this was not just an effect of the additional self-administration sessions. Therefore, we suggest that future studies explore this by directly comparing the development of binge-like METH intake between continuous access and varying intermittent access parameters, or by comparing cocaine and METH on the same intermittent access parameters.

Even though lifetime METH intake and reinstatement to METH-associated cues or a METH prime did not differ by adolescent stress exposure, isolation-housed rats demonstrated a greater sensitivity to reinstatement when exposed to a low dose of the pharmacological stressor yohimbine. This is consistent with previous work showing that stress exposure during adolescence sensitises female rats more so than males to later experiences with stress (Weintraub et al. 2010). Furthermore, this is consistent with unpublished findings from our group that chronic stress exposure during the early postnatal period in females, but not males, enhances the propensity to reinstate to METH-seeking behaviour when exposed to the same dose of yohimbine (0.625 mg/kg). Whilst no published studies have investigated the impact of adolescent social isolation on reinstatement to drug-seeking behaviour in females, a similarly designed study using male rats also reported that adolescent social isolation did not increase propensity to relapse on cue re-exposure or after a cocaine prime (Baarendse et al. 2014). However, in contrast to our findings, yohimbine-primed reinstatement was not altered by adolescent social isolation in male rats (Baarendse et al. 2014). This provides further support that females show a sensitised stress response when they have a history of chronic stress exposure. This may also help explain the underlying mechanism driving this sex-dependent effect. A yohimbine injection increases noradrenaline release (Szemeredi et al. 1991) and noradrenaline release facilitates HPA activation (Ma and Morilak 2005). Considering that female rats exposed to adolescent social isolation stress show a more pronounced HPA axis response to acute stress relative to males (Weintraub et al. 2010), yohimbine-induced noradrenaline release may elicit a stronger reinstatement effect in females through their hyper-reactive HPA axis. An additional consideration is that basal and stress-induced noradrenaline activity or receptor expression could also be impacted by adolescent social isolation in females; however, this has not yet been studied. This highlights that future studies should investigate how sex-specific differences in stress system reactivity and the noradrenaline system impacts vulnerability for stress-induced reinstatement to psychostimulant use.

The consideration of the DSM-5 criteria for substance use disorder in the implementation of schedules in the IVSA model used in this study is advantageous as it likely increases the translation of our findings. As most studies using an IVSA model include only one or a few of the schedules used in this study, the present work provides a more comprehensive understanding of the vulnerability to develop addiction symptoms following adolescent stress exposure. However, the implementation of the DA/NDA schedule as a means of examining persistence in drug-seeking had an unexpected effect on drug-taking behaviour in the socially isolated females, as well as not adequately capturing the targeted behaviour. A more appropriate schedule for measuring habitual or compulsive drug-taking would have been the application of a foot shock on receiving a METH infusion. This schedule would have measured compulsive drug-seeking despite punishment or adverse consequences (Deroche-Gamonet et al. 2004; Jonkman et al. 2012). Future work examining the impact of adolescent social isolation on drug addiction should implement this schedule.

Taken together, the results of this study demonstrate that negative adolescent experiences and different METH access conditions can increase sensitivity to METH and the development of an addiction phenotype in females. These findings highlight that further work bridging the gap in understanding sex-divergent responses to stress and drugs of abuse is needed.
